# Supplementary material for: Crystal structure of an adenovirus virus-associated RNA
Source: Nat Commun. 2019 Jun 28;10:2871. doi: 10.1038/s41467-019-10752-6 (PMC6599070; doi:10.1038/s41467-019-10752-6)
Supplement: Supplementary file 1 — Supplementary Information [file 41467_2019_10752_MOESM1_ESM.pdf]

Supplementary Information

Crystal structure of an Adenovirus Virus-Associated RNA

Hood et al.

Supplementary Figures 1-15
Supplementary Table 1
Supplementary Movie 1
Supplementary References

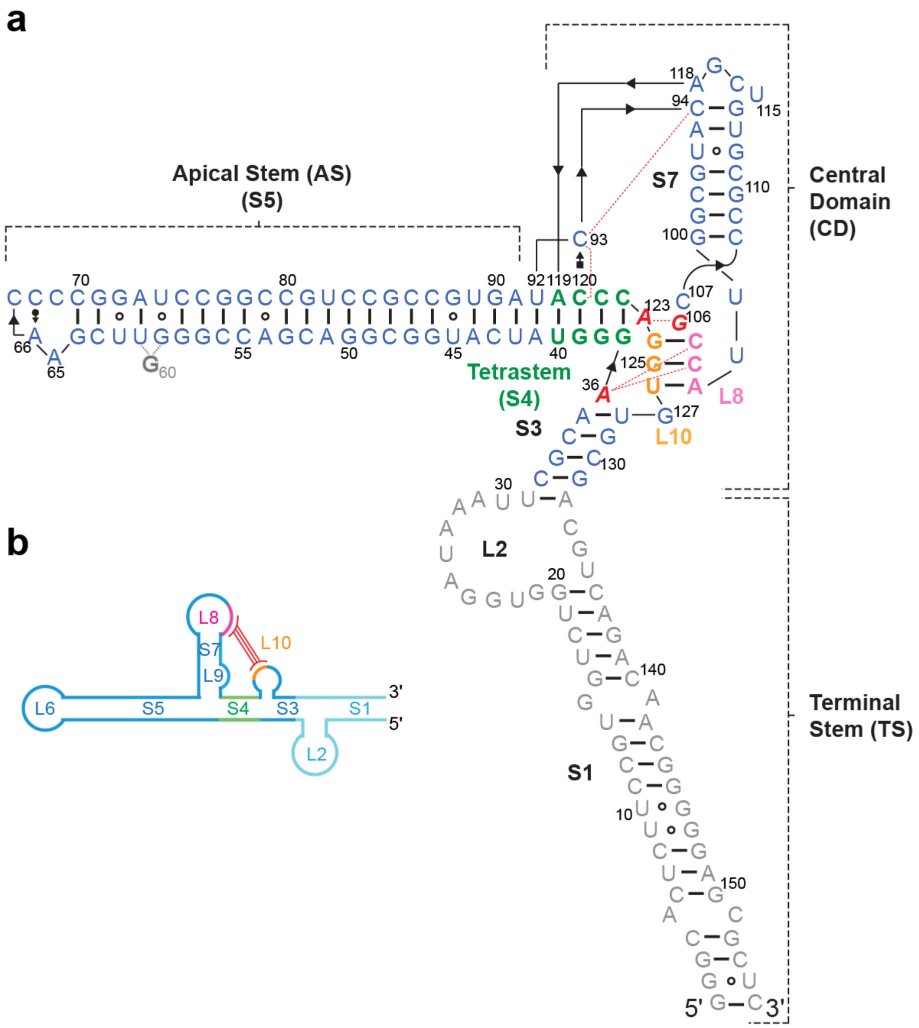

Supplementary Figure 1. Domain Organization and Secondary Structure of Adenovirus 2 Full-length VA-I RNA (**a**) Revised full-length VA-I RNA secondary structure based on the crystal structure. Stem 1 and Loop 2, which are absent from the crystal structure, are shown in gray. (**b**) Cartoon diagram of the conventional VA-I secondary structure.

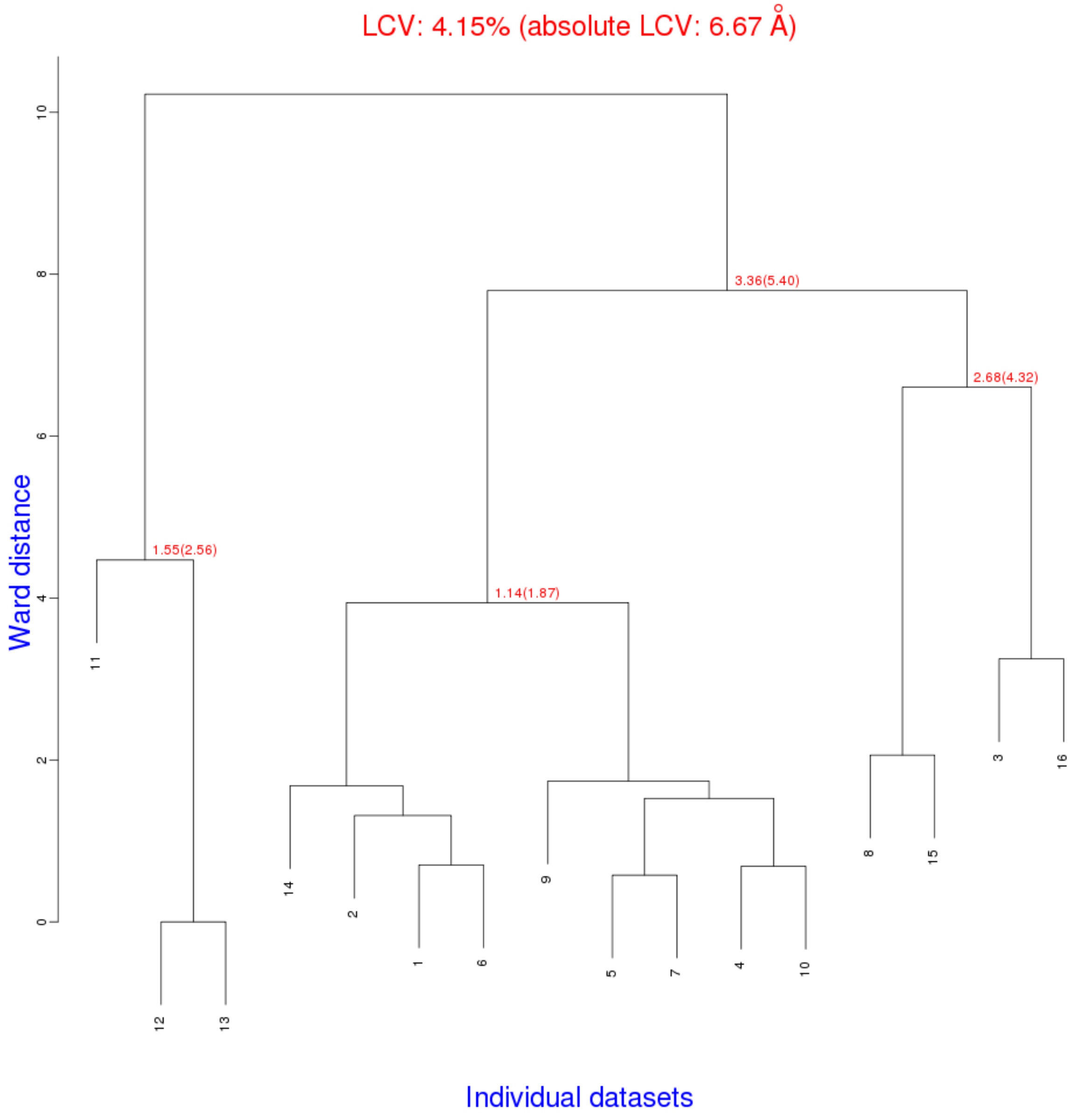

Supplementary Figure 2. Dendrogram of Crystal Unit Cell Variations Among 16 datasets.

The central cluster consisting of 9 datasets exhibited good isomorphism, with a linear cell variation (LCV) of 1.1% or 1.9Å, was used for multi-crystal averaging and phasing[1, 2, 3]. Crystallographic statistics of the 9 used datasets and merged phasing dataset are shown in Supplementary Table 1.

a

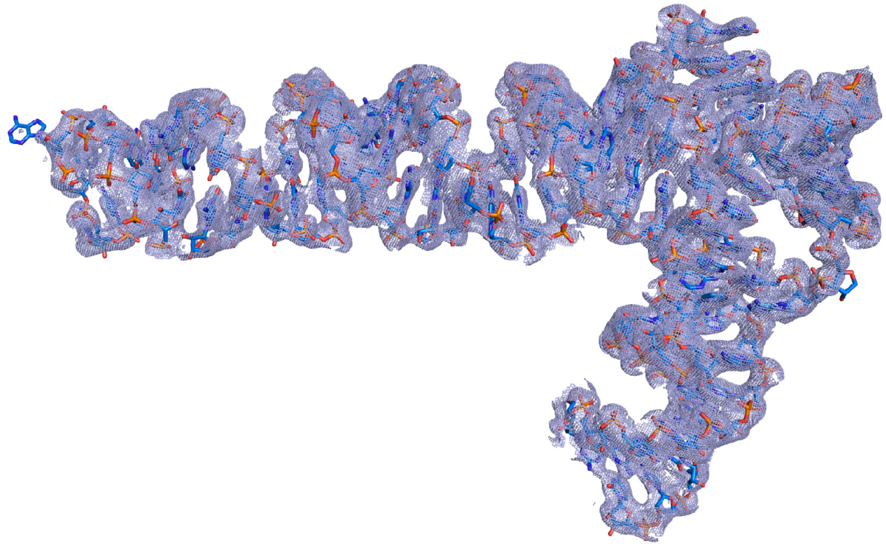

b

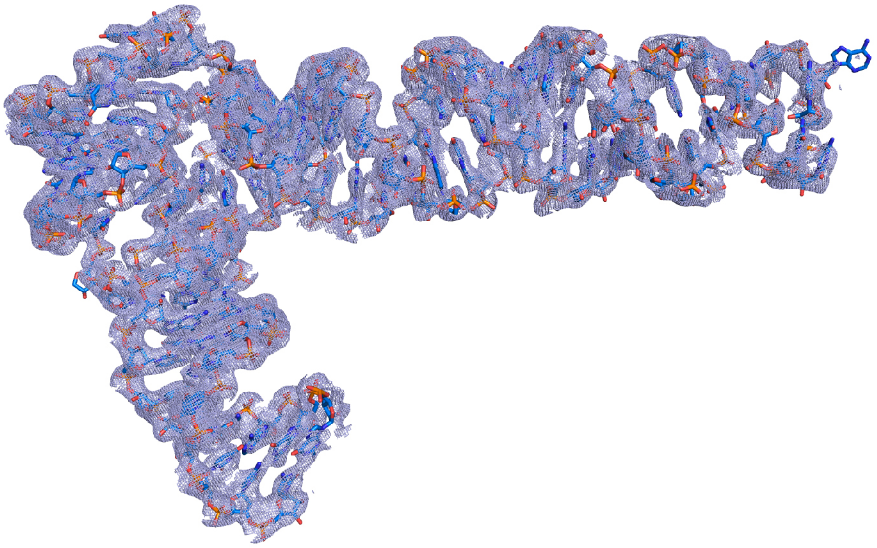

Supplementary Figure 3. Composite Simulated Anneal-omit 2|Fo|-|Fc| Electron Density Map Calculated Using the Final Model (1.0 s.d.) in Front (a) and Back (b) Views.

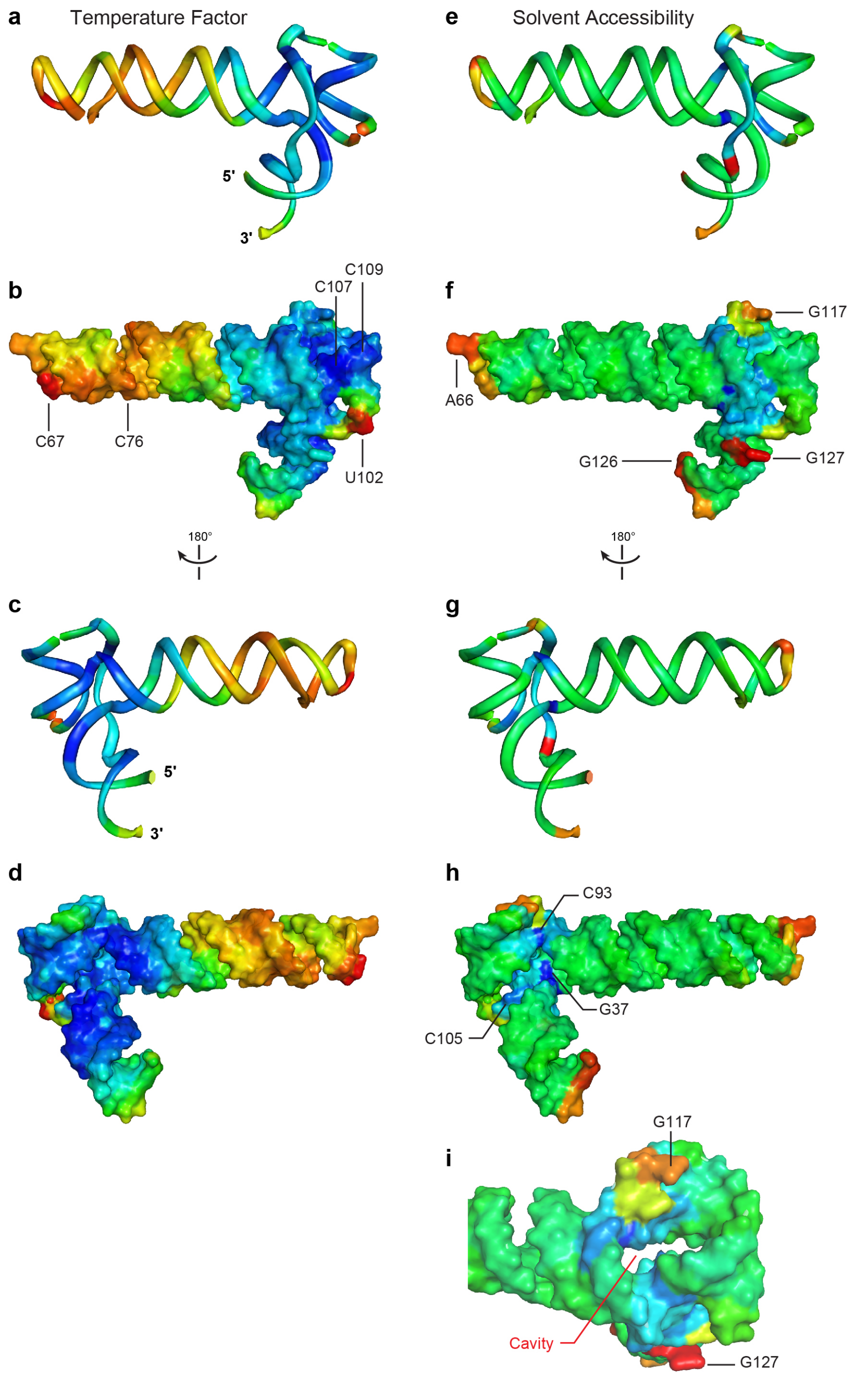

Supplementary Figure 4. Distribution of Temperature Factors and Solvent Accessibility on the VA-I RNA Surface. (**a-d**) Temperature factor distribution in backbone illustration (a and c) and surface representation (b and d) of VA-I RNA reveal a stable central domain (blue) and more flexible apical and terminal regions (green and yellow). (**e-h**) Solvent accessible surface of VA-I RNA in backbone illustration (e and g) and surface rendering (f and h). Most of the central domain remains accessible to the solvent. (**i**) A cavity in the central domain.

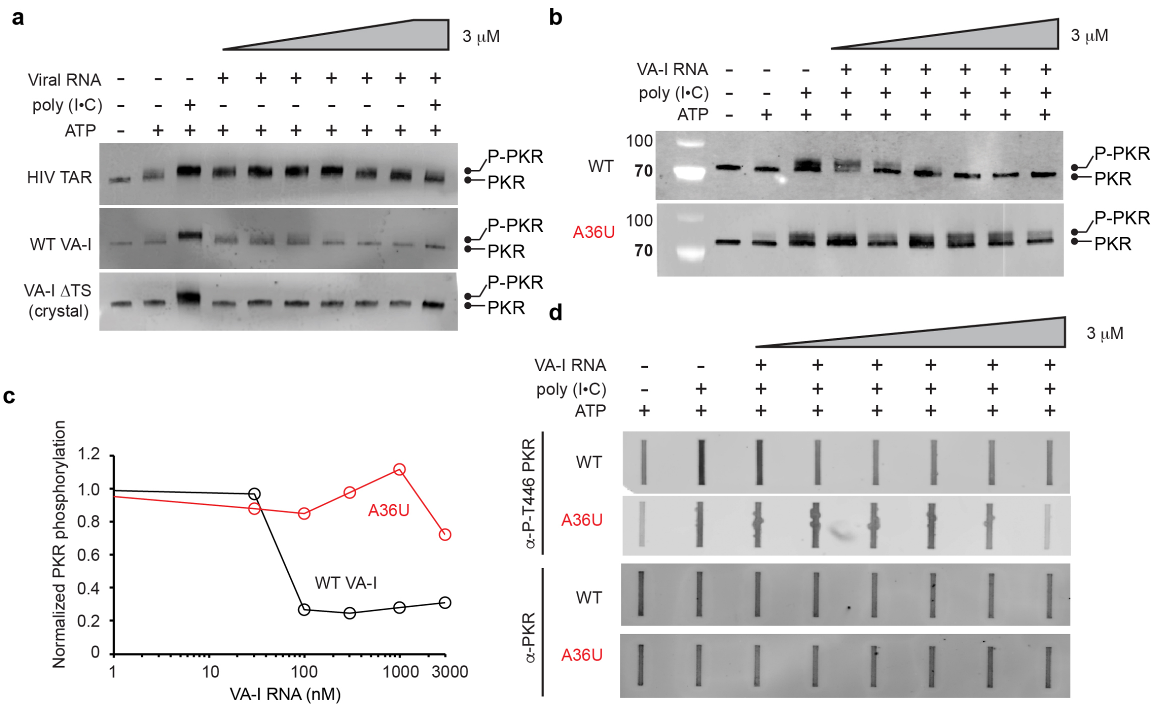

Supplementary Figure 5. PKR Activation and Inhibition Assays. (**a**) PKR activation assays as analyzed by immunoblotting using anti-phospho-Thr446-PKR[4,5], with HIV-I TAR (upper panel), WT VA-I (middle panel), and VA-I ΔTS (crystal construct, lower panel). Significant phosphorylation of PKR leads to reduced mobility on SDS-PAGE. While the HIV-I TAR and poly (I•C) activated PKR, VA-I RNAs did not. Six viral RNA concentrations were used: 10, 30, 100, 300, 1000, and 3000 nM. (**b**) PKR inhibition assays. PKR was pre-incubated with WT or mutant VA-I RNA and was subsequently challenged by the addition of poly (I•C) and ATP. (**c**) Quantitation of (b). (**d**) Representative Slot Blots for higher-throughput PKR inhibition assays[6]. See Methods for details.

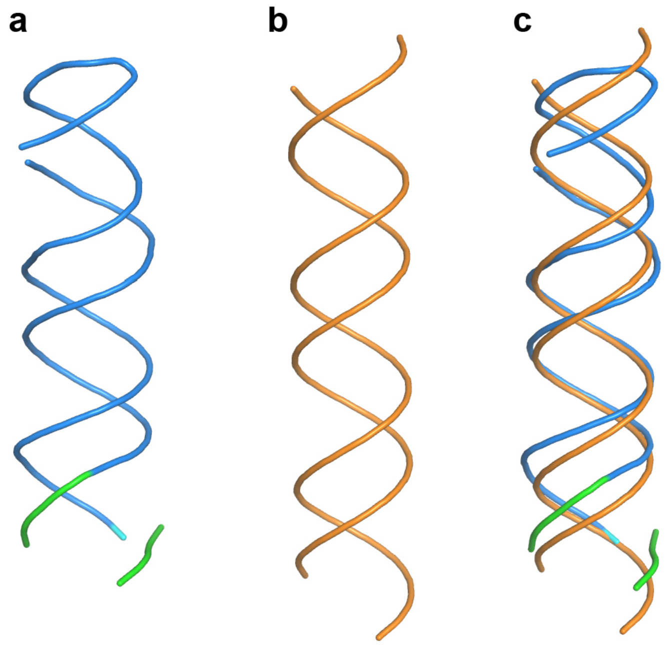

Supplementary Figure 6. Comparison of VA-I Apical Stem with an Ideal A-form dsRNA. RNA backbone trajectories of VA-I apical stem (**a**, blue), a 30-bp idealized A-form dsRNA (**b**, orange), and their overlay (**c**) show the deviation of VA-I apical stem geometry from regular A-form. VA-I apical stem is underwound, shorter, and wider than idealized dsRNA.

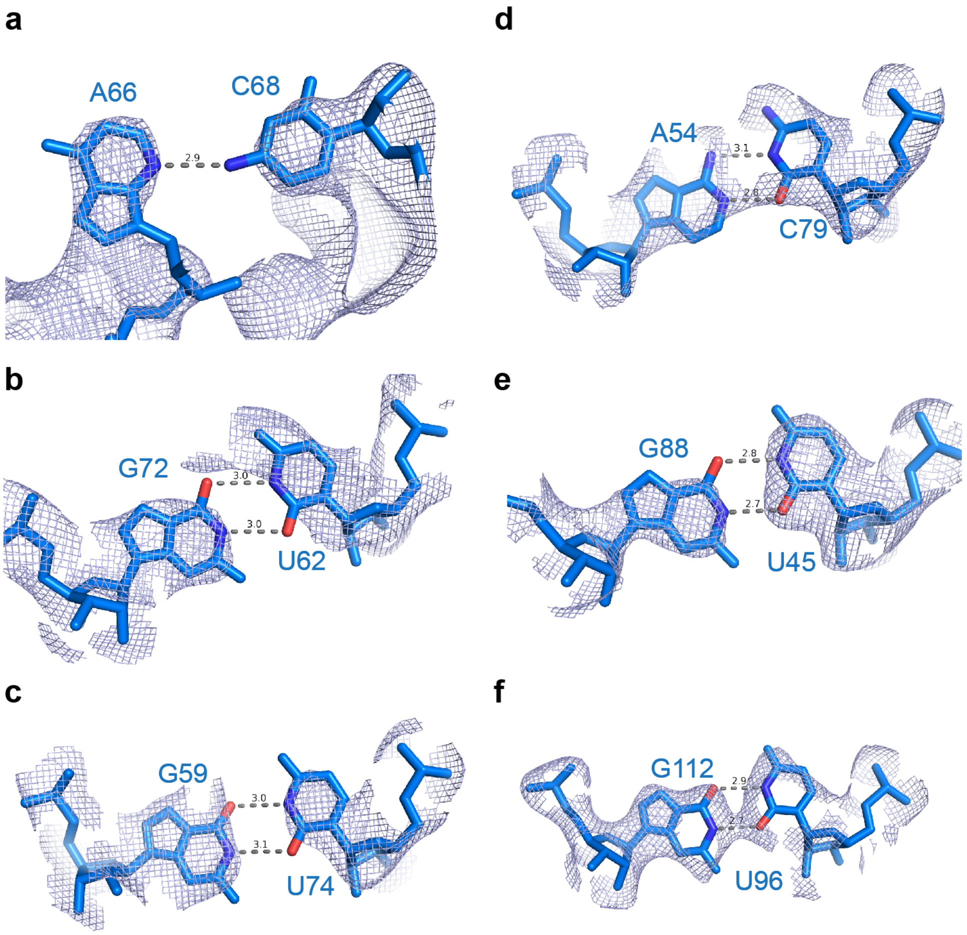

Supplementary Figure 7. Over-representation of Wobble Base Pairs in the VA-I Structure. (**a**-**f**) Six non-canonical base pairs are shown with their surrounding composite simulated anneal-omit 2|Fo|-|Fc| electron density calculated using the final model contoured at 1.0 s.d. Proposed hydrogen bonds are shown as gray dashes and estimated distances indicated in Ångströms. Note that 5 out of the 6 non-canonical base pairs are wobble base pairs.

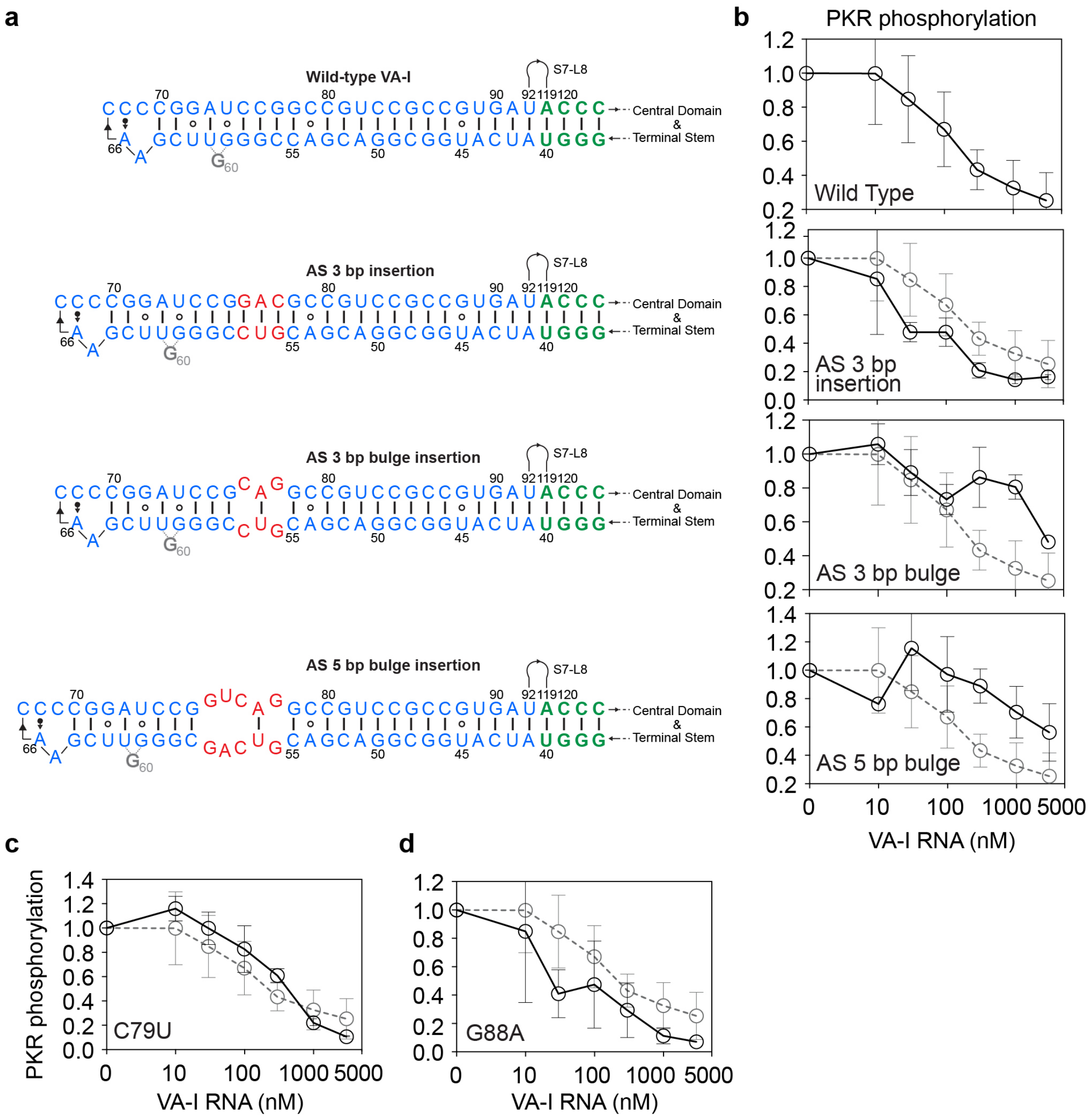

Supplementary Figure 8. Secondary Structures and PKR Inhibitory Activities of Additional Mutant VA-I RNAs.

(**a**) Secondary structures of WT VA-I and three apical stem (AS) insertion mutants. Positions of alterations are shown in red. (**b**) The WT VA-I titration curve. It is also shown as gray dotted lines for comparison in subsequent panels. Extending the apical stem (AS) by 3 bp slightly enhanced VA-I activity while inserting a 3-bp or 5-bp bulge in the AS reduced PKR-inhibitory activity. (**c** and **d**) Substitutions of individual wobble pairs by Watson-Crick pairs had minor effects on PKR inhibition, similar to the AS wobble-free mutant (Fig. 2e, bottom panel). C79U substitution replaces the $A^{+}54$•C79 wobble pair with a A-U pair (c); G88A substitution replaces the U45•G88 wobble pair with a U-A pair (d). Error bars represent s.d. from 3 independent replicates.

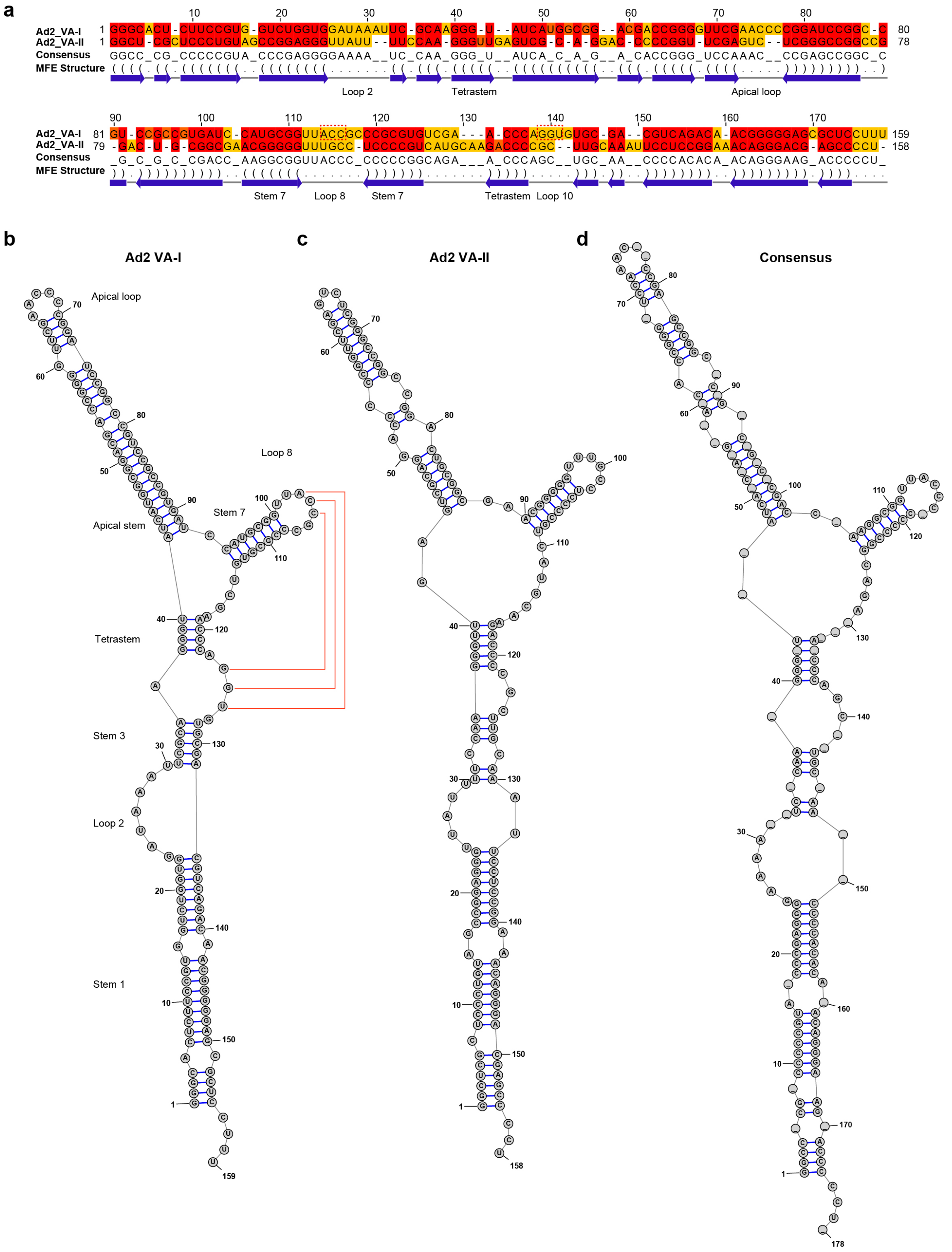

Supplementary Figure 9. Structural Alignment of Ad2 VA-I and VA-II

(**a**) Sequence and secondary structure alignment of Ad2 VA-I and VA-II. The pseudoknot-forming nucleotides in VA-I are boxed. The consensus and Minimum Free Energy (MFE) structure are indicated. (**b-d**) Secondary structures of Ad2 VA-I (b, based on the crystal structure; red lines denote pseudoknot formation), VA-II (c), and their consensus secondary structure (d) by Dynalign II[7].

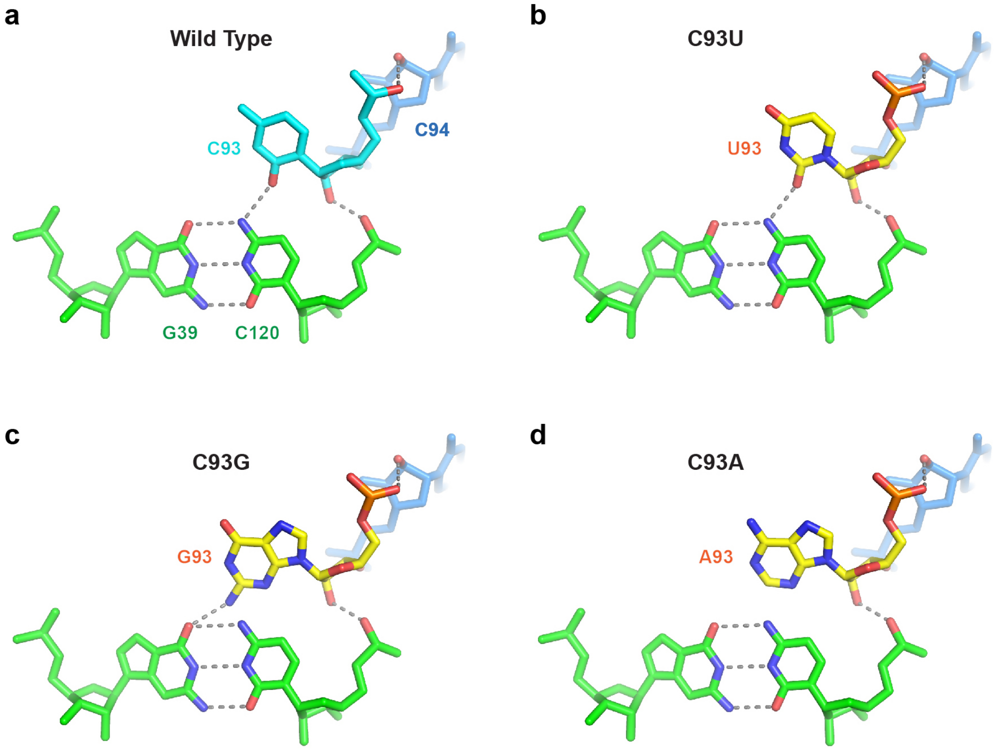

Supplementary Figure 10. ***In silico*** **substitutions of C93.**

(**a**) Hydrogen bonding patterns of the G39-C120•C93 base triple. The exocyclic 4-amino group of C120 makes a *cis* Hoogsteen-sugar-edge hydrogen bond (2.8 Å) to the 2-carbonyl group of C93. The G39-C120 base pair is part of the tetrastem and shown in green. (**b**, **c**, **d**) *In silico* substitutions of C93 (without geometric refinement) show that while C93U (b) and C93G (c) maintain similar interactions with the tetrastem, C93A lacks both the 2-carbonyl and 2-amino groups and is unable to bond with G39-C120 pair, leading to structural destabilization and significant defect in PKR inhibition.

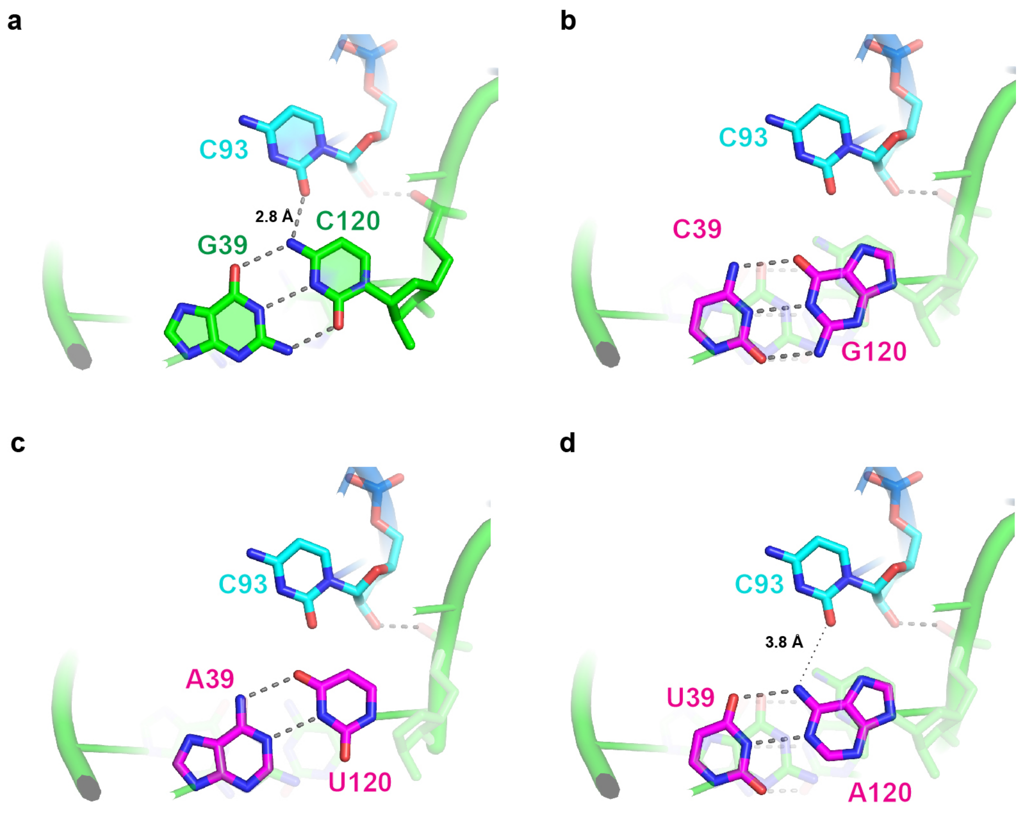

Supplementary Figure 11. Base Triple Formation Partly Drives the Sequence Conservation of the Tetrastem. (**a**) Hydrogen bonding patterns of the G39-C120•C93 base triple. The G39-C120 base pair is part of the tetrastem and indicated in green. (**b-d**) *In silico* substitution of the G39-C120 pair with a C-G (b), A-U (c) or U-A (d) pair does not support formation of the base triple. In (d), the exocyclic 6-amino group of A120 is located too far (3.8 Å) from the 2-carbonyl group of C93 to form a stable hydrogen bond. Formation of the base triple may impose base identity requirements in addition to maintaining the secondary structure within the tetrastem, and explain in part the high degree of sequence conservation.

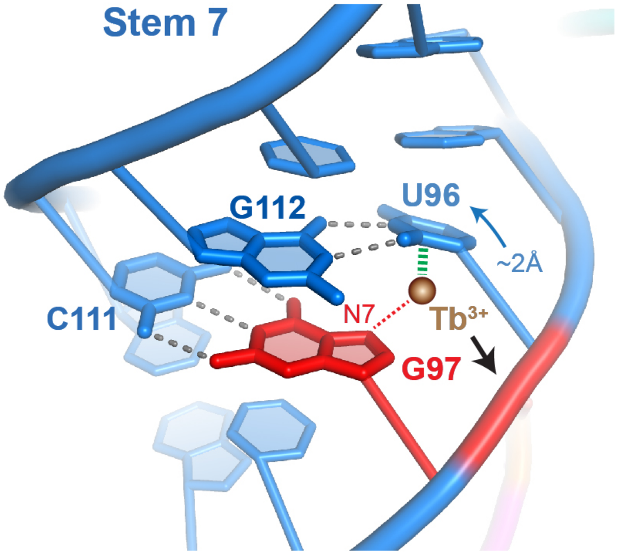

Supplementary Figure 12. Structural Basis of G97 Region's Exceptional Tb^{3+} sensitivity. The U96•G112 wobble pair shifts U96 base by ~2 Å outward into the major groove, which is well positioned to engage a cation-π interaction (green dashes) to stabilize a modelled Tb^{3+} (brown sphere) bound to the N7 edge of G97 (red dashes). In addition, the more flexible wobble pair backbone also facilitates in-line cleavage enhanced by Tb^{3+} ions[6].

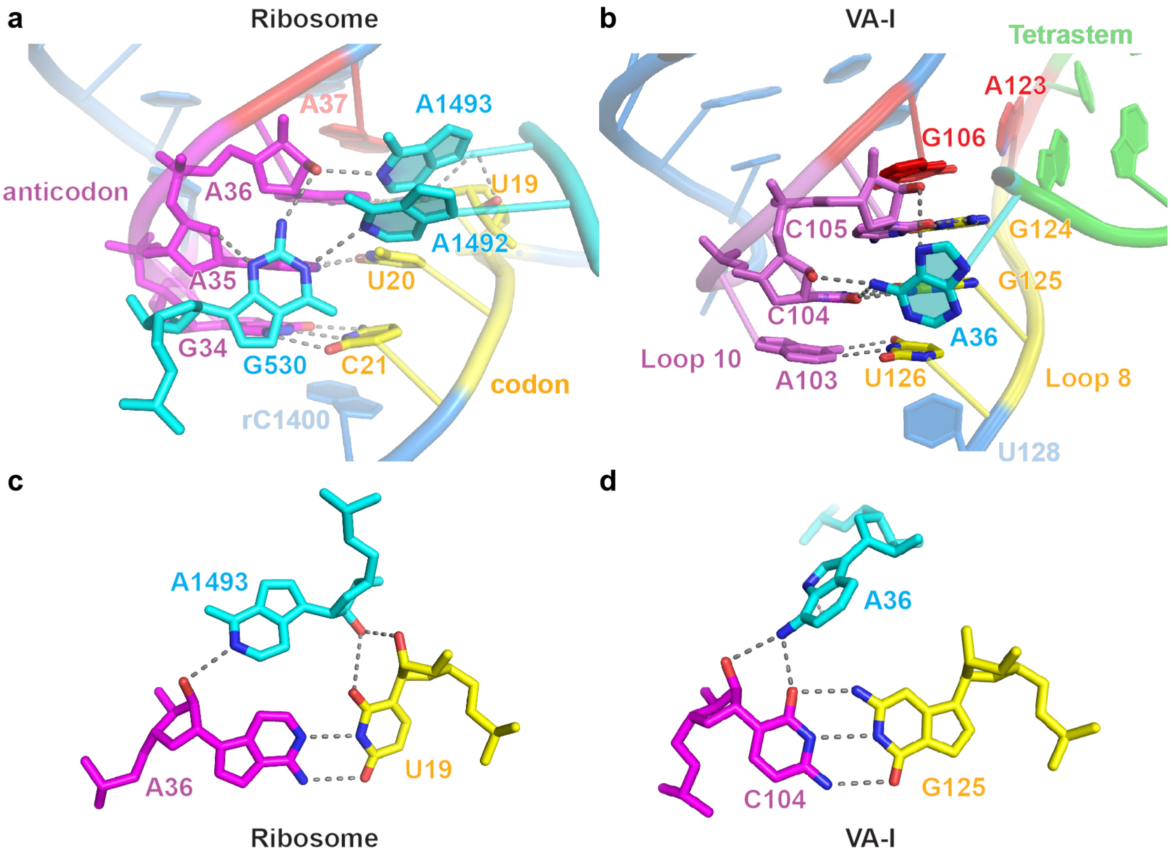

Supplementary Figure 13. Structural Comparison of A-minor Interactions in the Ribosome and Similar Interactions in VA-I RNA Central Domain.

(**a**) To ensure decoding fidelity in the ribosome A site, 30S ribosomal RNA (rRNA, cyan) imposes Watson-Crick geometry on the codon-anticodon interactions by extrahelically flipping A1492 and A1493 and shifting the G530 latch to interact with the minor groove of the codon-anticodon duplex (PDB: 4V5D)[8, 9, 10]. (**b**) In VA-I, A36 (shown in cyan in this figure for clarity) makes 3 hydrogen bonds to C104 and C105 in the minor groove in a similar manner. Like the three rRNA purines, A36 makes extensive hydrogen bonds to the first and second positions but not the wobble-like position (A103-U126) of the 3-bp helix (Fig. 4e-f). (**c**) The 2' OH of A1493 makes a bifurcated hydrogen bond with both the 2' OH and O2 of the nucleobase of the first nucleotide of the codon (U19). (**d**) In VA-I, the exocyclic amine (N6) of A36 makes similar interactions to C104 as in (c).

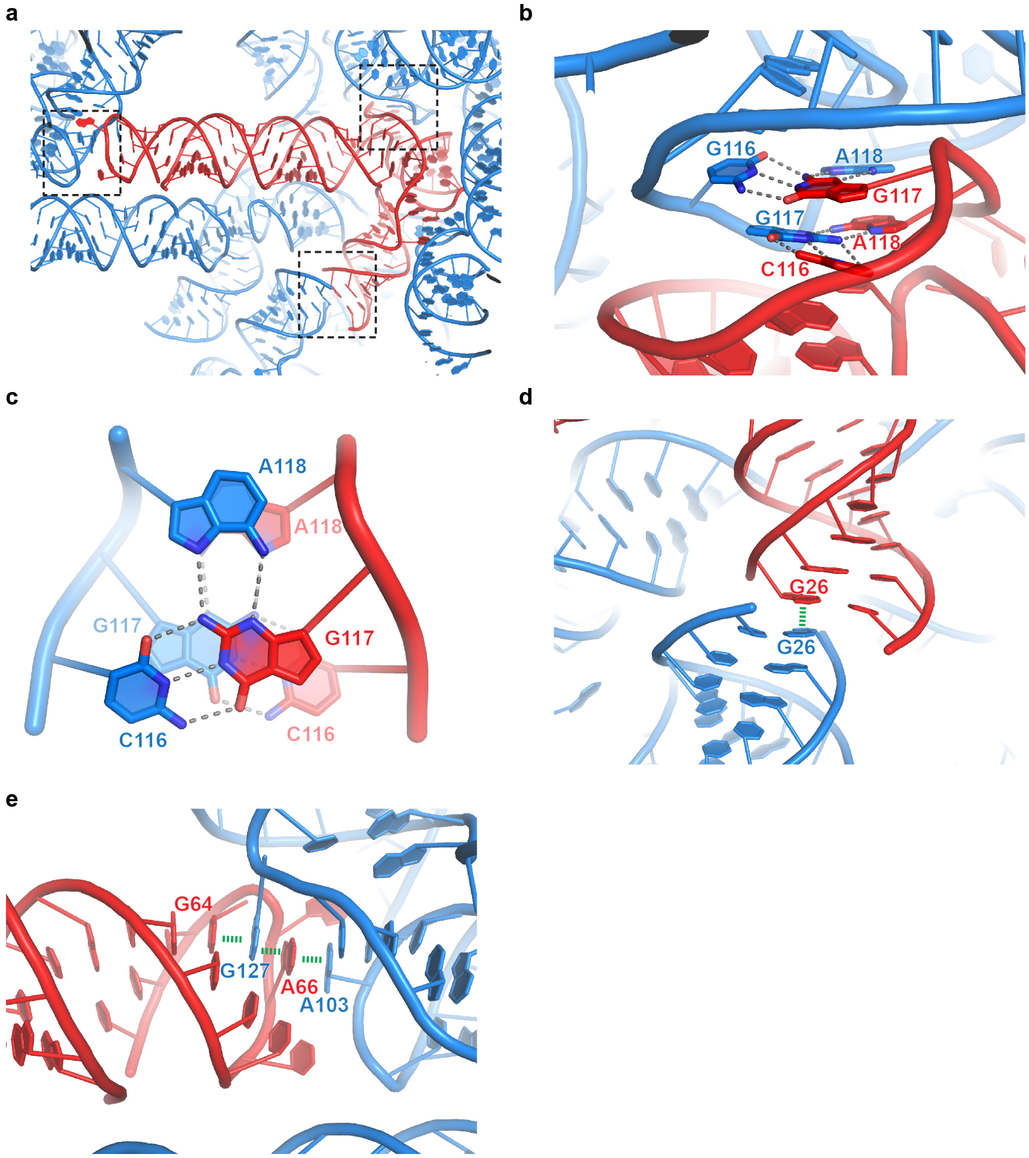

Supplementary Figure 14. Crystal-packing Interactions in the VA-I RNA crystals.

(**a**) Overall crystal-packing interactions. A reference VA-I molecule is shown in red while its symmetry-related molecules are in blue. (**b**) Packing between the top portion of Stem 7 and its symmetry mate. (**c**) Details of the base triple formation at the packing interface in (b). (**d**) Packing at the terminal stem. (**e**) Intercalation of G127 into the stacking gap between G64 and A66 in a symmetry-related molecule.

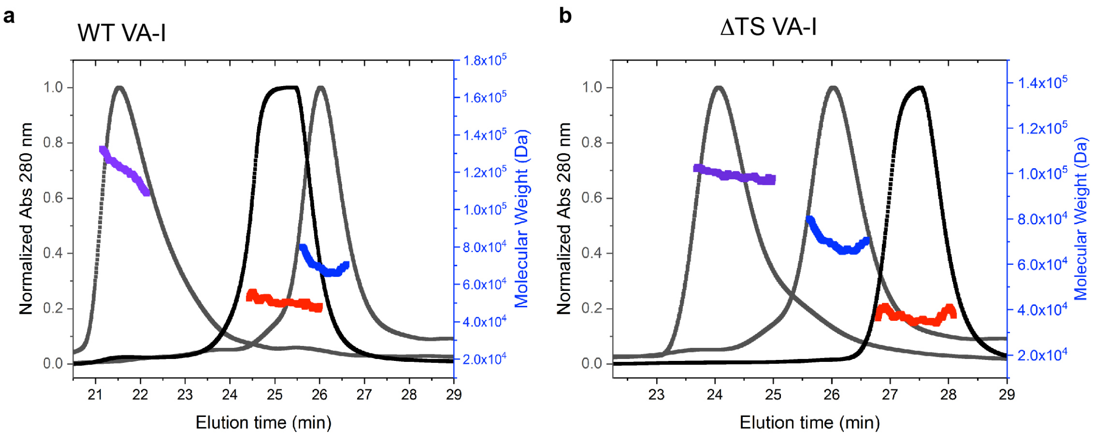

c

Sample	Expected M.W. (kDa)	Measured M.W. (kDa)	Stoichiometry
PKR	66.4	67.7 ± 1.24	-
WT VA-I	51	52.9 ± 1	-
ΔTS VA-I	32	33.9 ± 0.9	-
PKR+ WT VA-I	117.4	120 ± 8	1:1
PKR+ΔTS VA-I	98.4	100.2 ± 4.5	1:1

Supplementary Figure 15. Binding stoichiometry of PKR and VA-I analyzed by SEC-MALS.

(**a-b**) Size-exclusion chromatography profiles and molecular mass measurements of PKR complexes with wild-type (a) and ΔTS (b)VA-I RNAs. (**c**) Comparison of the expected and measured molecular masses and stoichiometries.

Supplementary Table 1. Summary of Crystallographic Statistics of Individual and Merged Datasets Containing Ir(III) Hexammine.

	Crystal 1	Crystal 2	Crystal 4	Crystals 5	Crystal 6	Crystal 7	Crystal 9	Crystals 10	Crystal 14	Merged Crystals 1,2,4,5,6,7,9,10,14
**Data collection**										
Space group	$P4_122$	$P4_122$	$P4_122$	$P4_122$	$P4_122$	$P4_122$	$P4_122$	$P4_122$	$P4_122$	$P4_122$
Cell dimensions										
a, *b*, *c* (Å)	100.7, 100.7, 131.2	100.8, 100.8, 130.3	101.3, 101.3, 131.0	101.0, 101.0, 131.4	101.3, 101.3, 132.1	101.3, 101.3, 132.1	101.3, 101.3, 130.3	101.3, 101.3, 130.3	101.2, 101.2, 130.5	101.0,101.0,130.7
α, β, γ (°)	90, 90, 90	90, 90, 90	90, 90, 90	90, 90, 90	90, 90, 90	90, 90, 90	90, 90, 90	90, 90, 90	90, 90, 90	90, 90, 90
Resolution (Å)	100.0-3.25 (3.33-3.25)	100.0-3.38 (3.47-3.38)	100.0-4.00 (4.10-4.00)	100.0-3.64 (3.73-3.64)	100.0-3.60 (3.69-3.60)	100.0-3.98 (4.08 - 3.98)	100.0-3.65 (3.74 - 3.65)	100.0-3.38 (3.47 - 3.38)	100.0-4.02 (4.12 - 4.02)	101.0-4.05 (4.53-4.05)*
R_{merge} (%)	21.9 (114)	13.6 (62.3)	26.7 (72.6)	19.1 (73.5)	19.8 (68.2)	18.6 (66.8)	15.4 (64.0)	14.4 (84.1)	13.0 (58.5)	43.2 (157)
$\langle I \rangle / \langle \sigma(I) \rangle$	11.0 (2.9)	15.2 (4.6)	12.7 (6.4)	12.7 (4.6)	14.3 (5.6)	13.2 (5.0)	13.7 (4.8)	13.9 (3.6)	11.7 (4.5)	34.2 (21.2)
Completeness (%)	99.9 (100.0)	99.9 (100.0)	99.9 (100.0)	99.9 (100.0)	100.0 (100.0)	99.8 (100.0)	99.9 (100.0)	99.9 (100.0)	99.9 (100.0)	100.0 (100.0)
Redundancy	14.2 (14.8)	14.2 (14.7)	17.1 (17.8)	14.1 (14.7)	13.4 (14.2)	17.1 (17.9)	14.1 (14.6)	14.1 (14.7)	9.2 (9.7)	123.7 (127.9)
CC1/2	1.00 (0.83)	1.00 (0.95)	1.00 (0.96)	1.00 (0.94)	1.00 (0.97)	1.00 (0.93)	1.00 (0.95)	1.00 (0.90)	1.00 (0.92)	1.00 (1.00)
Ano CC1/2	0.57 (0.03)	0.70 (0.11)	0.66 (0.19)	0.64 (0.22)	0.73 (0.21)	0.69 (-0.04)	0.69 (0.13)	0.69 (0.05)	0.67 (0.12)	0.95 (0.80)

Values in parentheses are for the highest resolution shell.

Supplementary References

1. Foadi J*, et al*. Clustering procedures for the optimal selection of data sets from multiple crystals in macromolecular crystallography. *Acta Crystallogr D Biol Crystallogr* **69**, 1617-1632 (2013).

2. Liu Q*, et al*. Structures from anomalous diffraction of native biological macromolecules. *Science* **336**, 1033-1037 (2012).

3. Akey DL, Terwilliger TC, Smith JL. Efficient merging of data from multiple samples for determination of anomalous substructure. *Acta Crystallogr D Struct Biol* **72**, 296-302 (2016).

4. Dzananovic E*, et al*. Impact of the structural integrity of the three-way junction of adenovirus VAI RNA on PKR inhibition. *PLoS One* **12**, e0186849 (2017).

5. Yuen KC, Xu B, Krantz ID, Gerton JL. NIPBL Controls RNA Biogenesis to Prevent Activation of the Stress Kinase PKR. *Cell Rep* **14**, 93-102 (2016).

6. Wilson JL, Vachon VK, Sunita S, Schwartz SL, Conn GL. Dissection of the adenoviral VA RNAI central domain structure reveals minimum requirements for RNA-mediated inhibition of PKR. *J Biol Chem* **289**, 23233-23245 (2014).

7. Fu Y, Sharma G, Mathews DH. Dynalign II: common secondary structure prediction for RNA homologs with domain insertions. *Nucleic Acids Res* **42**, 13939-13948 (2014).

8. Korostelev A, Trakhanov S, Laurberg M, Noller H. Crystal structure of a 70S ribosome-tRNA complex reveals functional interactions and rearrangements. *Cell* **126**, 1065-1077 (2006).

9. Selmer M*, et al*. Structure of the 70S ribosome complexed with mRNA and tRNA. *Science* **313**, 1935-1942 (2006).

10. Ogle JM, Brodersen DE, Clemons WM, Jr., Tarry MJ, Carter AP, Ramakrishnan V. Recognition of cognate transfer RNA by the 30S ribosomal subunit. *Science* **292**, 897-902 (2001).
