# Supplementary material for: Crystal structure of an adenovirus virus-associated RNA
Source: Nat Commun. 2019 Jun 28;10:2871. doi: 10.1038/s41467-019-10752-6 (PMC6599070; doi:10.1038/s41467-019-10752-6)
Supplement: Supplementary file 3 — Description of Additional Supplementary Files [file 41467_2019_10752_MOESM3_ESM.pdf]

Description of Additional Supplementary Files

File Name: Supplementary Movie 1
Description: A 360° View of the Adenovirus VA-I RNA Crystal Structure. The VA-I RNA is shown in blue, with the Tetrastem in green, Loop 8 in violet, Loop 10 in yellow, and the three helix-capping purines A36, G106 and A123 in red.
